# Rheology, Texture and Swallowing Characteristics of a Texture-Modified Dysphagia Food Prepared Using Common Supplementary Materials

**DOI:** 10.3390/foods12122287

**Published:** 2023-06-06

**Authors:** Xin Wang, Liyuan Rong, Mingyue Shen, Qiang Yu, Yi Chen, Jinwang Li, Jianhua Xie

**Affiliations:** State Key Laboratory of Food Science and Resources, Nanchang University, Nanchang 330047, China; wx163ylfz@163.com (X.W.); rongliyuan01@163.com (L.R.); shenmingyue1107@ncu.edu.cn (M.S.); yuqiang8612@163.com (Q.Y.); chenyi-417@163.com (Y.C.); lijw202418@163.com (J.L.)

**Keywords:** dysphagia, IDDSI, swallowing, rheological property, sensory evaluation

## Abstract

A dysphagia diet is a special eating plan. The development and design of dysphagia foods should consider both swallowing safety and food nutritional qualities. In this study, the effects of four food supplements, namely vitamins, minerals, salt and sugar, on swallowing characteristics, rheological and textural properties were investigated, and a sensory evaluation of dysphagia foods made with rice starch, perilla seed oil and whey isolate protein was carried out. The results showed that all the samples belonged to foods at level 4 (pureed) in The International Dysphagia Diet Standardization Initiative (IDDSI) framework, and exhibited shear thinning behavior, which is favorable for dysphagia patients. Rheological tests showed that the viscosity of a food bolus was increased with salt and sugar (SS), while it decreased with vitamins and minerals (VM) at shear rates of 50 s^−1^. Both SS and VM strengthened the elastic gel system, and SS enhanced the storage modulus and loss modulus. VM increased the hardness, gumminess, chewiness and color richness, but left small residues on the spoon. SS provided better water-holding, chewiness and resilience by influencing the way molecules were connected, promoting swallowing safety. SS brought a better taste to the food bolus. Dysphagia foods with both VM and 0.5% SS had the best sensory evaluation score. This study may provide a theoretical foundation for the creation and design of new dysphagia nutritional food products.

## 1. Introduction

Dysphagia is considered to be a swallowing disorder in that food fails to pass smoothly down the esophagus from the oral cavity to the stomach, which can limit food intake and nutrient absorption [1]. In the case of newborns and the elderly who are prone to swallowing disorders, in particular, it greatly impacts their normal lives and growth [2]. An unformed food bolus or the misclosure of the pharyngeal epiglottis during oral processing caused by poor chewing and swallowing abilities increase the risk of malnutrition, choking and pneumonia in dysphagia patients [3,4]. The prevalence of dysphagia is about 13% in people over 65 years old and about 51% in institutionalized elderly [5]. Along with increased population aging, dysphagia is affecting more and more elderly in terms of their quality of life, and mental and physical health. Actually, this swallowing disorder frequently bothers patients with postoperative muscle loss, neurological impairment and Alzheimer’s disease, resulting in additional time and expense required for patient treatment [6,7]. Therefore, it is necessary to work on dysphagia foods.

Thicker products such as pastes and purees are often considered more suitable for dysphagia patients because of the delayed flow of liquid and more time for safe swallowing [8]. Softer, more uniform, easier to chew and more elastic foods seem to take fewer oral efforts to swallow [9,10]. Thus, to meet these needs of special populations, the modification of food texture by adding hydrocolloids such as starches and proteins has received attention. For instance, beef patties obtained a softer texture with tapioca starch added [11], and carrot puree showed a better viscosity with xanthan gum added [12], which was more suitable for dysphagia patients. The International Dysphagia Diet Standardization Initiative (IDDSI), was established worldwide to provide better dietary recommendations for individuals with dysphagia [13]. A series of easy-to-run tests (flow test, fork drip test, spoon tilt test, etc.) are used by the IDDSI framework to evaluate the dysphagia-relevant characteristics based on food texture and flow thickness, classifying all foods from fully liquid to fully solid on a scale of 0~7, a total of 8 levels [14]. Dysphagia foods prepared with mung bean starch and flaxseed protein as gel with calcium salts added were classified as level 5 (minced and moist) and level 6 (soft and bite-sized) foods in the IDDSI framework [15]. In addition, the desirability and quantity of dysphagia foods can be increased by optimizing taste and shape, for instance by adding small doses of salt to mask the fishy flavor of fish sauce with increased chewiness and elasticity [16], and 3D printing to enhance the attractiveness and interest of thickened foods made from black fungus [17].

The texture-modified products may be poorer in nutrients and more likely to result in malnutrition and muscle weakness than ordinary foods [18,19]. A total of 18.6% of elderly with dysphagia are undernourished [20]. Energy and protein intakes were both lower in dysphagia patients receiving the texture-modified diet than those treated with a normal hospital diet [21]. For dysphagia patients, the food is usually thickened by adding hydrocolloids such as starch, xanthan gum, and guar gum as thickening agents to suit their compromised swallowing ability, thus reducing the nutrient density of original food [22]. In addition, dysphagia diets are generally deficient in fruits, vegetables and whole grains, which leads to a demand for nutritional diversity of foods for dysphagia patients [22]. Nutritional intake deficiencies occur from the energy intake of basic nutrients such as carbohydrates and proteins and the absorption of micronutrients such as vitamins and minerals [23]. Compared to vegetable oils, perilla seed oil is known to contain up to 67% α-linolenic acid, which can well meet the unsaturated fatty acid requirements of the elderly today, as well as lowering blood lipids, improving memory, liver protection and other effects that are among the reasons behind its popularity [24]. On the other hand, in Britain’s aging population, vitamin B_12_ deficiency accounts for 12% and folic acid deficiency for 15% of elderly [25], and 67.8% of the over 65s have varying degrees of vitamin and mineral inadequacy [23]. Furthermore, a vicious loop is created when malnutrition exacerbates dysphagia through neuromuscular dysfunction [26]. Due to a single nutritional pattern, using dysphagia food prepared by combining carrot puree with xanthan gum and κ-carrageenan gum may make it challenging to satisfy the nutritional requirements of patients [12]. Therefore, the design of dysphagia food products should also consider the wide range of food matrices and nutritional properties [7].

More and more attention is being paid to the dysphagia population and suitable dysphagia foods. Currently, the development of most dysphagia products is focused on improving the texture properties of natural foods, which could lead to inadequate nutritional composition and single-product patterns. In this paper, the most fundamental ingredients (rice starch, whey isolate protein and perilla seed oil) were used to construct a dysphagia food bolus model based on nutritional requirements that gives more design possibilities with regard to swallowing foods. The effects of common supplements (vitamins, minerals, salt and sugar) on the swallowing characteristics and texture properties were investigated. Moreover, the correlations between rheology, texture and sensory evaluation were explored to provide a theoretical basis for developing more practical and nutritional dysphagia foods.

## 2. Materials and Methods

### 2.1. Materials and Chemicals

Rice starch (RIS) (≥99%) was obtained from Wuxi JinNong Biology Science and Technology Co. (Wuxi, China). Whey isolate protein (WPI) (protein, 89%; moisture, 4.7%; ash, 2.7%; fat, 1.3%; and lactose, 0.1%) was bought from Hilmar Corporation (Hilmar, LA, CA, USA). Perilla Seed Oil (PSO) (≥99%) was purchased from KangShanYuan Fats & Oils Co. (Nanchang, China). Salt and sugar were obtained from Tianhong supermarket (Nanchang, China). Mixed vitamins and mixed minerals were obtained from RuiPu Biotechnology Co. (Tianjin, China). The materials above were all food grade. All of the trials were conducted with Millipore ultrapure water.

### 2.2. Sample Preparation

#### 2.2.1. The Design of a Simple Dysphagia Food System

RIS, WPI and PSO were used as carbohydrate, high quality protein and fatty acid suppliers, respectively and their energy coefficients were 17 kJ/g, 17 kJ/g and 37 kJ/g, respectively. The ratio of flour to water was 1:5, in each 100 g of material powder, containing PSO (X g), RIS (Yg) and WPI (Z g). According to the General Rules for Formula Food for Special Medical Purposes (GB 29922-2013) [27] and the Chinese Dietary Reference Intakes (2013) [28] published by the Chinese National Health and Family Planning Commission, dysphagia food boluses were designed to satisfy the following requirements:

(i) No less than 295 kJ energy per 100 g of ready-to-eat products:

37X + 17Y + 17Z ≥ 295 × (1 + 5);(1)

(ii) At least 0.7 g protein per 100 kJ energy:

Z ≥ [(37X + 17Y + 17Z)/100] × 0.7;(2)

(iii) A total of 50–65% energy from carbohydrates and 20–30% energy from fats:

50% ≤ 17Y/(37X + 17Y + 17Z) ≤ 65%,(3)

20% ≤ 37X/(37X + 17Y + 17Z) ≤ 30%.(4)

According to Equations (1) and (4), the PSO content is 9.73~16.45%. To simplify the formulation and calculation, PSO content was set to 10% and 15% for follow-up design.

According to Equations (2) and (3), the contents of RIS and WPI were set as follows:

A: When the PSO content is 15%, the RIS content is 58.82~76.47%, and the WPI content is 14.00~26.18%. To simplify the formulation and calculation, the RIS and WPI contents were set to 60% and 25%, 65% and 20%, and 70% and 15%, respectively.

B: When the PSO content is 10%, the RIS content is 55.88~72.65%, the WPI content is 13.30~34.12%. To simplify formulation and calculation, the RIS and WPI contents were set to 60% and 30%, 65% and 20%, and 70% and 15% respectively.

The dysphagia food bolus components are shown in Table 1.

#### 2.2.2. The Design of the Dysphagia Food Bolus with Supplementary Materials

According to the National Food Safety Standard Determination of General Rules for Formula Food for Special Medical Purposes (GB 29922-2013) [27], the vitamin, mineral, salt and sugar contents were added by relying on Table 2 and Table 3. Because the weight of the supplementary materials is very small, the additional contents are on top of the RIS, PSO and WPI total weight.

The corresponding proportions of substances were weighed and dissolved in water with stirring to obtain suspensions, which were then heated in a magnetic stirring water bath from room temperature to 95 °C for another 10 min to produce mud-like dysphagia food bolus.

### 2.3. Water-Holding Capacity (WHC)

The WHC of the food sample was estimated by the centrifugation method [29]. Samples weighing 5 g were centrifuged at 10,000× *g* for 10 min at room temperature and the supernatants were removed.

WHC (%) = (m_2_ − m_0_)/(m_1_ − m_0_) × 100%, (5)

where m_0_ is the weight of the empty tube, m_1_ is the total weight of the sample and tube before centrifugation, and m_2_ is the total weight of the precipitate and tube after centrifugation.

### 2.4. IDDSI Test Methods

Definitive IDDSI levels of the food bolus were determined by the fork drip test and spoon tilt test [30]. Fork drip test: the samples were held up and examined to see if they would trickle through the tines or prongs of a fork. Spoon tilt test: the states of samples were observed when the spoon was placed steadily and tilted sideways, along with the appearance of the spoon after they slid off.

### 2.5. Rheological Characterization

In order to better emulate the effects of oral shear on the rheology and viscoelasticity of the food bolus, the samples were equilibrated in a water bath at 37 °C (human body temperature) for 2 h. Then, the sample was set up between parallel plates (40 mm diameter and 1.0 mm gap) equipped with clamps and finally measured by a DHR-2 rheometer (TA Instruments, New Castle, DE, USA) [28].

#### 2.5.1. Steady-State Viscous Flow Tests

Flow sweep was used in shear rates (*γ*) of 0.1–100 s^−1^ with 1% strain to study the variation of viscous flow behavior, and the flow curves were fitted with a power-law model (*η* = *Kγ^n^*), where consistency *K* (Pa·s*^n^*), the flow behavior index (*n*) and the apparent viscosity (*η*) at 0.1, 1, 10, 50 and 100 s^−1^ of samples were recorded [31].

#### 2.5.2. Small Amplitude Oscillatory Shear Tests

The viscoelastic changes of the dysphagia food bolus were evaluated by the storage modulus (*G*′) and loss modulus (*G*″), which were examined using an oscillatory frequency model in the frequency range of 0.1 Hz–16 Hz with 1% strain [6,31,32,33].

### 2.6. Back Extrusion Test for Swallowing the Food Bolus

The texture properties of the samples were performed using a texture analyzer (TA-XT plus, Stable Micro System Co., London, UK) for the back extrusion test after equilibrating at 37 °C for 2 h [34]. Parameter settings: cylindrical probe was P/36R, the pre-test, test and post-test speeds were 1 mm/s, compression deformation was 15%, and trigger force was 5 g.

### 2.7. Structural Characteristics Tests

#### 2.7.1. Scanning Electron Microscopy (SEM)

A freeze-dried food bolus was fixed and sprayed on the sample stage with gold via a JFC-1600 ion sputtering device (JEOL Ltd., Tokyo, Japan). The gold-sprayed sample was observed using SEM (JSM6701F, Tokyo, Japan) with 5 kV accelerating voltage, and digital images were captured using XT Microscope-Control software (version FESEM 1.0, JEOL Ltd., Tokyo, Japan) [35].

#### 2.7.2. X-ray Diffraction (XRD) Analysis

Freeze-dried samples were tested at 40 kV and 40 mA with Cu Kα radiation using an X-ray diffractometer (D8 Advance, Bruker, Karlsruhe, Germany). The determined range was from 5° to 40° with a scanning rate of 1 °/min [36].

#### 2.7.3. Confocal Laser Scanning Microscopy (CLSM)

The food bolus (500 mg) was dissolved in 10 mL ultrapure water to prepare the suspension. Nile red solution (0.01% in methanol, *w/v*) and Nile blue A solution (0.01% in methanol, *w/v*) were first mixed in a ratio of 2:1 as the dyeing agent. Then, a 1 mL suspension was mixed with 40 μL dyeing agent for 10 min. The CLSM images were observed by confocal laser scanning microscopy (Leica TCS SP8, Leica Microsystems GmbH, Wetzlar, Germany) with excitation wavelengths of 488 nm and 633 nm, respectively [16].

### 2.8. Color Measurements

The boluses were laid flat on the bottoms of dishes after being balanced at 37 °C for 2 h, and color parameters (lightness, L*; redness/greenness, a*; yellowness/blueness, b*; chroma, C_ab_*; hue, h_ab_*; and color variations, ΔE*) were recorded and calculated using a portable colorimeter (HP-2136, Puxi, Shanghai, China) [37].

C_ab_* = [(a*)^2^ + (b*)^2^]^1/2^ (6)

h_ab_* = arctan(b*/a*) (7)

ΔE* = [(ΔL*)^2^ + (Δa*)^2^ + (Δb*)^2^]^1/2^ (8)

### 2.9. Sensory Evaluation

The sensory evaluation conformed with the ethical and testing requirements in the national standard (GB/T 10220-2012) published by the Standardization Administration of China [38]. A team of 6 students aged 20–25 years, majoring in food science and having undergone courses in sensory analysis, took part in the study as panelists. Before sensory testing, the evaluation participants were trained in dysphagia, dysphagia foods and scoring criteria for each indicator to ensure that the evaluation results were informative [10,34,37]. Samples were kept at 37 °C for evaluation, and water was provided for oral rinsing after each sample was tested to avoid bias [16]. The evaluation criteria for sensory evaluation were referred from the studies by Ribes et al. [37] and Xie et al. [16], and color, organization, taste, flavor, adhesion and swallowing were selected (Table 4). The scores of every level were referred from the study by Xie et al. [16]. In order to emphasize the swallowing characteristics of the food bolus, the scores of each criterion were modified, especially the percentage of the swallowing score in the total score, which was increased. 

### 2.10. Statistical Analysis

Data are expressed as mean ± SD for at least triplicate determinations, while IBM SPSS Statistics (version 26.0, Chicago, IL, USA) and OriginPro (version 9.0, Stat-Ease Company, Northampton, MA, USA) software were used for statistical analysis, and one-way ANOVA was used for intergroup analysis, followed by Duncan’s method for post hoc multiple comparisons, with *p* < 0.05 considered as significant difference.

## 3. Results

### 3.1. Construction of the Dysphagia Food System

#### 3.1.1. WHC and IDDSI Level of the Dysphagia Food System 

**Figure 1:**
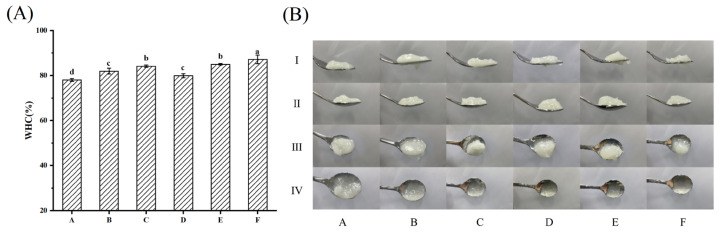
The water holding capacity (**A**) of gels used to construct the dysphagia food system. The different letters are significantly different (*p* < 0.05). IDDSI tests (**B**) of gels used to construct the dysphagia food system. A–F are prepared dysphagia food bulus rely on Table 1. The fork driptest (Ⅰ raw) and spoon tilttest (Ⅱ, Ⅲ and Ⅳ raws corresponding before, during and aftertilting the spoon).

The mixed suspensions of RIS, WPI and PSO were heated to form different textural dysphagia food boluses by cross-linking interactions between each other and the water molecules [39]. Under the heating process, starch molecules absorb water and expand, the crystalline structures are broken, and on cooling, starch molecules are rearranged to form a stable gel system [29]. Proteins and oils can be present in the gel network formed by the rearrangement of starch molecules to form starch–protein complexes, starch–oil complexes or starch–protein–oil complexes [40]. Evaluation of the physicochemical properties of potato-starch-based foods and their interactions with milk protein and soybean oil was carried out. When the RIS content was definite, comparing samples A and D, samples B and E, and samples C and F, it was found that WPI could give better WHC to the swallowed food bolus than PSO, which may be due to the fact that proteins with more hydrophilic groups are more likely to cross-link with starch to form more hydrogen bonds, and possess better hydrophilicity than hydrophobic oils [40] The food bolus showed the best water holding capacity when RIS was 70%, PSO was 10% and WPI was 20% (Figure 1A). The better WHC endowed the dysphagia food bolus with a softer texture and more lubrication with less resistance in accessing the esophagus and stomach [16].

From the fork drip test (Figure 1(B-Ⅰ)), all samples accumulated over the fork top and formed a fishtail shape underneath but did not drip off. In the spoon tilt test (Figure 1 (B-Ⅱ~Ⅳ)), the samples were able to stack well on the spoon and slowly slide off when tilted, indicating that all six samples may be classified as level 4 (pureed) of the IDDSI framework [14]. Samples D, E and F slipped from the spoon with only a very small residue and a clear film appeared on the spoon surface, while samples A and B had much larger residues, which suggested that boluses A (PSO:RIS:WPI = 15%:60%:25%) and B (PSO:RIS:WPI = 15%:65%:20%) could be highly susceptible to residual food debris in the pharynx, with a strong risk of accidental aspiration during swallowing [1].

#### 3.1.2. Rheological Properties

**Figure 2:**
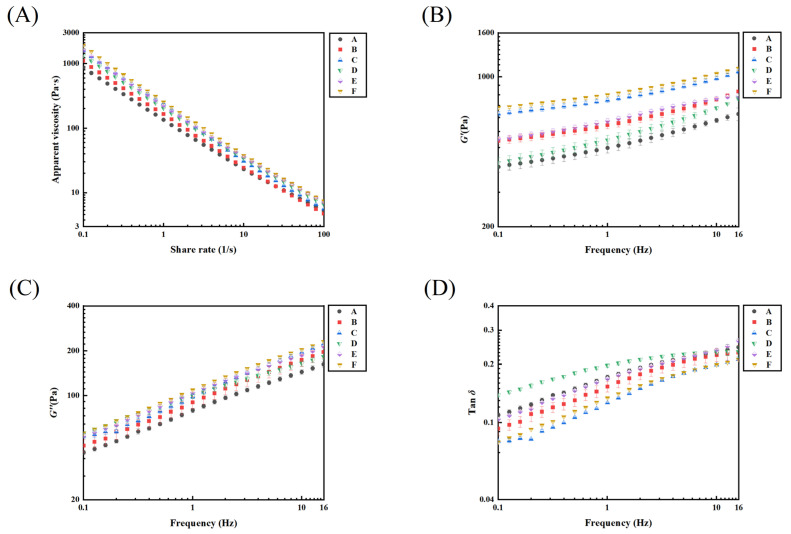
Apparent viscosity (**A**), storage modulus (*G*′) (**B**), loss modulus (*G*″) (**C**) and loss factor (Tanδ) (**D**) curves of the dysphagia food system.

As shown in Figure 2, the apparent viscosity of all samples decreased with increasing shear rate, exhibiting a shear thinning behavior, which was suitable for dysphagia patients to slow down the swallowing process [41]. Sample F (PSO:RIS:WPI = 10%:70%:20%) exhibited the highest apparent viscosity (*η*) and also a lower flow behavior index (*n* = 0.193 ± 0.005), indicating more pseudoplastic behavior and better safety of swallowing [42].

The loss factor Tanδ is the ratio of loss modulus (*G*″) to storage modulus (*G*′), which indicates the viscoelastic characteristics [42]. When the food Tanδ is higher than 1, it is harder to control for people with poor chewing and swallowing abilities, because the food tends to be more liquid and its flow rate is higher and difficult to control, so that it could easily enter the airway to cause choking and coughing [41]. For dysphagia patients, a soft gel with a Tanδ less than 1 is very suitable, not only because the flow rate is easy to control, but the food is more easily deformed and passed smoothly through the esophagus [41]. *G*′ is greater than *G*″ for all samples, which is the distinctive feature of dysphagia foods [43]. Similar viscoelastic patterns are shown in Figure 2B–D, but F exhibited the highest modulus values than the others, especially *G*′. This shows the high elasticity property of food bolus F. The Tanδ value also indicates the energy loss of the food during chewing and compression [43] Among all samples, F had the smallest Tanδ, indicating that F could require less energy to be consumed during chewing and compression, which is easier for dysphagia patients. In order to make the swallowing of the food bolus safer, the F bolus with 70% RIS, 10% PSO and 20% WPI was chosen to continue the investigation of the effects of the supplementary materials on the texture and swallowing characteristics.

#### 3.1.3. The CLSM of the Food Bolus

**Figure 3:**
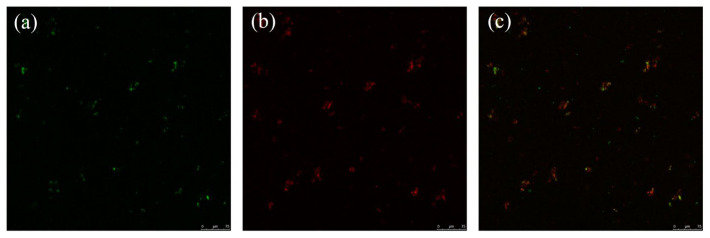
The CLSM images of food bolus F. From left to right are WPI staining with Nile blue A (**a**), WPI staining with Nile red (**b**), and the combination of the two (**c**), respectively.

In order to verify the feasibility of the food bolus construction, CLSM was used to observe the food bolus structure, especially the distribution of oils and proteins, as the content of both was lower than that of starch. Proteins and oils were stained with Nile blue A and Nile red, respectively. Protein was marked in green (Figure 3a), and oil was marked in red (Figure 3b). From the results, the protein and oil could be uniformly dispersed in the gel system. In the combined image of them (Figure 3c), the common distribution of green and red indicates that the mixing distribution of protein and oil in food bolus F was relatively consistent, which also shows that the three materials could be well mixed in the starch-based system from the side [16]. The result indicates that the food bolus construction is successful.

### 3.2. Effects of Supplementary Materials on the WHC of the Food Bolus

**Figure 4:**
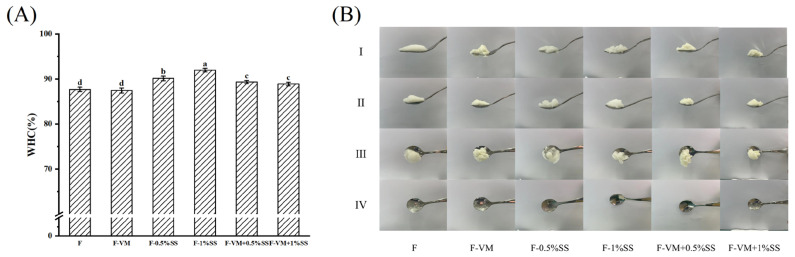
The WHC (**A**) of the food boluses with supplementary. The different letters are significantly different (*p* < 0.05). IDDSI tests (**B**) of the food boluses with supplementary materials. The fork driptest (Ⅰ raw) and spoon tilttest (Ⅱ, Ⅲ and Ⅳ raws corresponding before, during and aftertilting the spoon).

The effects of different supplementary materials on the WHC of the dysphagia food bolus are shown in Figure 4A. Compared to F, VM did not significantly affect the WHC of the food system (*p* < 0.05), but the 1% SS caused a clear increase from 87.69 ± 0.49% to 90.13 ± 0.53%. In the formative dysphagia gel system, salt ions might contribute to an enhanced hydrophobic interaction that blocked the migration of free water to trap more water in gel space [44]. It has been reported that sucrose could also enhance the WHC of the system by forming some small cavities in gels [45]. However, compared to F-SS, the WHC of F-VM-SS was reduced by the addition of VM, which could be attributed to the fact that the released Ca^2+^ and Mg^2+^ resulted from the presence of Cl^−^ replacing Na^+^. Furthermore, the Ca^2+^ increased the linkage between proteins and weakened the cross-linking among different molecules, leading to the aggregation of proteins and the formation of a rough gel structure, with a decrease in WHC [35].

### 3.3. Classification of the Dysphagia Food Bolus with Supplementary Materials

The results of the fork drip test and spoon tilt test for all samples based on the IDDSI framework are shown in Figure 4B. From the fork drip result, all samples could be well stacked on the top of the fork and there was no dripping from the slit. The samples could accumulate well above the spoon and slide slowly when tilted, indicating that the gels all belonged to level 4 (pureed foods) in the IDDSI framework [14]. However, after slipping down, there were still small residues of F-VM and F-VM + 1%SS sticking to the spoon surface, which is unsafe for dysphagia patients. After swallowing, less material still remained in the pharynx and did not enter the esophagus, leading to a more dispersed bolus, which could have a high probability of slipping into the trachea and causing serious aspiration [1]. Compared to the F bolus with a clear film on the spoon, F-0.5%SS, F-1%SS and F-VM + 0.5%SS were found to have better swallowing characteristics in that spoons were clearer and smoother after slipping off, making them more suitable for those suffering from dysphagia.

### 3.4. Rheological Analysis of the Effects of Supplementary Materials on the Food Bolus

#### 3.4.1. Flow Rheological Properties of the Food Bolus with Supplementary Materials

**Figure 5:**
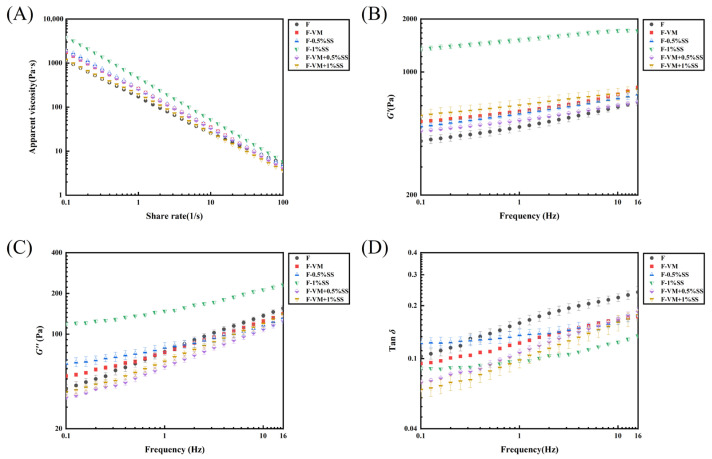
Apparent viscosity (**A**), storage modulus (*G*′) (**B**), loss modulus (*G*″) (**C**) and loss factor (Tanδ) (**D**) curves of the food boluses with supplementary materials.

Figure 5A shows the viscous flow behavior of samples with added excipient. At the shear rate of 50 s^−1^, which was simulated for normal human oral chewing, the apparent viscosities (*η*) of the food bolus were enhanced with SS, while decreased with VM (Table 5). However, the elderly or dysphagia groups have impaired chewing and swallowing abilities, which prevents the oral shear rate from increasing to 50 s^−1^ [46]. Herranz et al. rheologically characterized three commercial thickening products for dysphagia patients. They highlighted the importance of measuring the viscosity at low shear rates as a transient increase in apparent viscosity of the bolus is accompanied by the decrease in shear rate associated with the swallowing process [6]. The lower shear rates of 0.1, 1 and 10 s^−1^ were chosen to compare the *η* values of food samples. The addition of VM increased the *η* of the food system compared to F, while at 50 s^−1^, it decreased. This suggests that F-VM may be more suitable for dysphagia patients than F at lower shear rates, and also that F-VM could have stronger shear thinning. This may provide the possibility for people with varying degrees of swallowing problems to choose foods with the appropriate apparent viscosity and shear characteristics to suit them better [6] The food bolus with SS already added showed a similar tendency, where VM reduced the *n* at 50, 10, 1 and 0.1 s^−1^ shear rates. The strong water absorption of SS led to more water molecules present in systems with enhanced *η*, while the divalent mineral ions were involved in hindering intermolecular linkages resulting in a decreased *η* [16,35]. The flow behavior index (*n*) was obtained by fitting a power-law model to flow curves from six groups, and significant differences were observed (*p* < 0.05). The curves were in good agreement with R^2^ higher than 0.95 [47]. As shown in Table 5, all supplementary materials decreased the *n* values and increased the shear thinning behaviors of the gel system. The *η* of thickened products would be reduced to a very small value with the increasing shear rate at a lower *n* value, which did not facilitate swallowing [48]. Nevertheless, the samples in this study were maintained in the range of pudding-type food consistency (*K*) in the NDD standard even at higher shear rates, which could enable a safe swallowing process [49].

#### 3.4.2. Viscoelastic Properties of Food Boluses with Supplementary Materials

The curves of the dynamic flow properties of all samples are shown in Figure 5B–D. SS significantly increased the *G*′ and *G*″ of the samples in a dose-dependent manner (*p* < 0.05). However, VM was observed to reduce the modulus increases, which was consistent with the variation in steady-state flow characteristics. In the gel system of dysphagia foods, mineral ions might interact with starch and protein molecules through hydrogen bonds or van der Waals forces to modify their own mechanical properties. Similar conclusions were obtained when Ca^2+^ was used to act on jicama starches and proteins, reducing the viscosity and gel strength [35,50,51]. In addition, the *G*′ and *G*″ of all samples were increased with an increase in angular frequency, and were fitted with power-law functions (*G*′ = *G*_0_′f*n*′ and *G*″ = *G*_0_″f*n*″) to account for modulus dependence on frequency [6], where *n*′ and *n*″ denote the dependence of *G*′ and *G*″ on frequency (f), respectively. As shown in Table 5, SS induced a decrease in the *n*′ value of gels, resulting in a lower dependence of *G*′ on f, suggesting that SS could support stronger molecular interactions to promote more powerful gels [52]. The *n*′ values were similar to the nectar and pudding-like products for dysphagia [53]. The VM and SS had positive effects on the elastic properties of the prepared gel system, with *G*′_1Hz_ increased (Table 5). Compared to the bolus with SS alone, although F-VM-SS significantly reduced the *G*′ and *G*″ (*p* < 0.05), there was no significant difference in Tanδ, indicating that the equilibrium of the elastic–viscous system in the samples was not changed remarkably.

### 3.5. Effects of Supplementary Materials on the Mechanical Properties of the Food Bolus

The acceptability of dysphagia food is highly correlated with food texture. For dysphagia patients, better springiness, greater cohesiveness, higher softness and easier chewiness are preferred [54]. SS increased the springiness, chewiness and resilience of the food bolus (Table 6), which is beneficial for dysphagia patients, allowing food to pass smoothly through the pharynx into the esophagus. A high level of Na^+^ in salt could promote molecular connection and strong water absorption could promote the formation of gels with a more solid network structure [36,55], thus improving food texture. However, the positive effect of SS was weakened when all materials were present together. In a study of calcium-ion-induced *Mesona chinensis* polysaccharide–whey protein isolate gels, it was found that calcium ions increased protein–protein linkage, decreased protein–polysaccharide cross-linking, and weakened gel strength [35]. More divalent ions in VM were released to drown out Na^+^, which served as the bridge to strengthen cross-linking between the same molecules and weaken that between different molecules [53]. Compared with the SS-only food bolus, chewiness and resilience were reduced, but springiness was not significantly different, suggesting that F-VM+SS was still acceptable to dysphagia patients. The cohesiveness of the six groups was no different. Although SS and VM significantly increased the hardness and gumminess of the food bolus, they decreased when all supplementary materials were present at the same time. This suggests that the risk of accidental aspiration was likely to be minimal when both SS and VM were present, while the texture of the food bolus was likely to be the softest [4]. In addition, F-VM-1%SS showed a more pronounced decrease in texture than powders containing 0.5% SS (e.g., chewiness decreased from 271.5 ± 31.4 to 182.5 ± 7.0 at 0.5% added, and from 354.7 ± 28.5 to 211.3 ± 44.1 at 1% added). There was no significant difference in mechanical properties between the F-VM + 0.5%SS and F-VM + 1%SS boluses in the study, but a better textural property was still observed than without any supplementary materials.

### 3.6. Effects of Supplementary Materials on the Microstructure the Food Bolus

**Figure 6:**
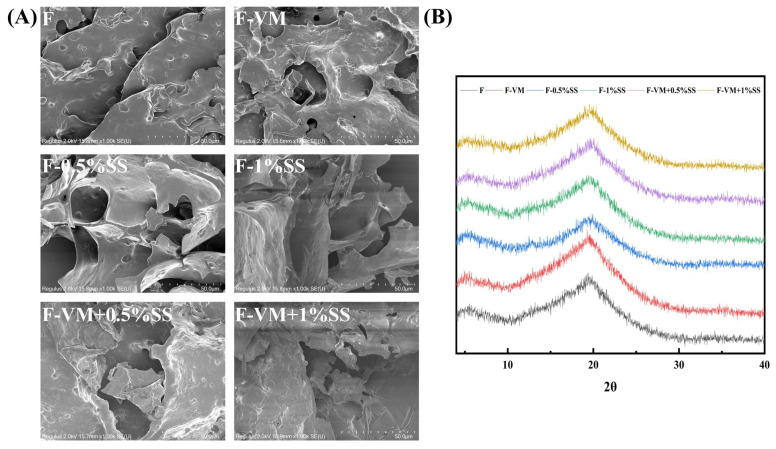
Scanning electron micrographs (**A**) and X-ray diffraction curves (**B**) of food boluses with supplementary materials.

SEM could provide better visualization of the interior structural changes in the samples [56]. The samples without any supplementary materials showed a laminar structure with one piece pressed on top of the other (Figure 6A). The addition of VM contributed to larger holes and erratic cross-linking (Figure 6A). After SS was mixed, a few regular gel networks could be clearly observed, giving the bolus a harder texture and better gel quality. In a study of the influence of Nacl on gel properties of *Mesona chinensis* polysaccharide–maize starches, it was found that Na^+^ contributed to forming honeycomb network structure gels and enhanced the interaction between starch and polysaccharide molecules through electrostatic interactions to form stronger gels [52]. Moreover, a rupture of gel structure was observed when adding VM to the system containing SS, which explains why the food softened when all supplementary materials were added. The presence of more divalent ions may induce aggregation between homo-molecules, while breaking bonds and connections between different molecules such as proteins and starches [53]. 

XRD is an effective method for evaluating the crystal structure of dysphagia food boluses [57]. The XRD patterns of dysphagia food were all similar, with a broader diffraction peak at 2θ of 20° owing to amorphous structures (Figure 6B). A very broad dispersion peak was shown between 2θ of 10°–30° in the XRD spectrum under the modified waxy maize starch with saturated fatty acid chlorides, which was attributed to the broken starch crystal structure finally being converted to an amorphous one [56]. The addition of supplementary materials did not affect the general view of the diffraction pattern of the gel systems.

### 3.7. Effects of Supplementary Materials on the Color Properties of the Food Bolus

**Figure 7:**
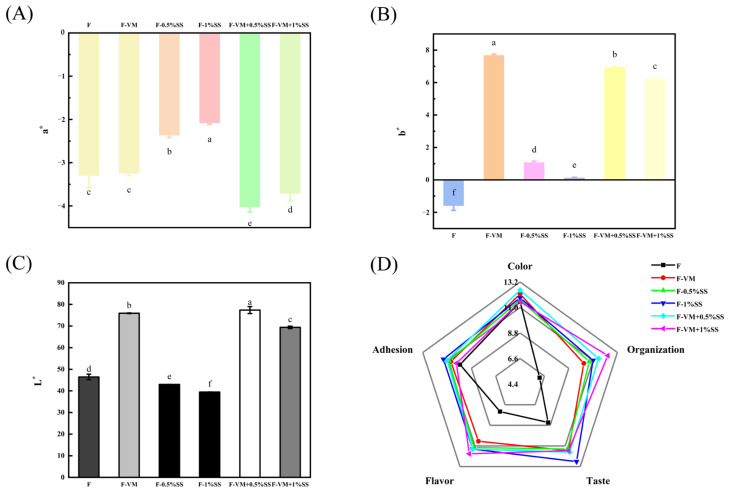
Redness/greenness, a* (**A**), yellowness/blueness, b* (**B**), lightness, L* (**C**) and average scores of sensory evaluations (**D**) of dysphagia food with supplementary materials. The different letters are significantly different (*p* < 0.05).

In relation to color analysis (Figure 7A–C and Table 7), the b* and C_ab_* values of the F-VM, F-VM + 0.5%SS and F-VM + 1%SS pellets were significantly higher than those of F (*p* < 0.05), and the ∆E* values were larger, most likely because various vitamins contained in VM added more yellow to the food bolus. In VM, VB_2_, also known as riboflavin, is bright yellow and exists stably under heating in neutral conditions, VC and VA powders are generally slightly yellow, VE is yellow-green, folic acid takes the form of yellowish crystals or flakes, and pantothenic acid is a yellowish sticky material [23]. In addition, nicotinic acid and VB_2_ are more stable under heating conditions, which could better maintain their nutritional properties [23]. The gel system with a network structure formed by the complexation of starch, protein, oil and water has a good protection effect on unstable VC, VE, folic acid and pantothenic acid. Proteins and oils could form a V-shaped wrapping structure through hydrogen bonding after starch molecules absorb water and expand, which could better wrap or load vitamins without being broken and protect the nutritional activity [58]. Soybean isolate protein and pectin particles formed a gel system with a definite network structure as a delivery system which could increase the encapsulation rate of VE up to 72.1 ± 5.9% and exhibit good in vitro antioxidant activity [58]. Adding VM together increased the L* of the system compared to adding SS alone to the food bolus. The L* value reflects the brightness of food. It may be attributed to the fact that the addition of VM gave the food F-SS a softer structure and a smaller particle size to scatter more light [59]. Fish sauce with repeated grinding possessed a smaller particle size and an increased specific surface area, which increased the brightness value of fish sauce by scattering more light [16]. Compared with F, in F-0.5%SS and F-1%SS it was found that SS could marginally reduce L*, b*, C_ab_* and h_ab_* values and their ∆E* values were also smaller at 5.325 ± 0.324 and 8.364 ± 0.140. However, SS did not affect color changing and the yellow color increase brought by VM to the food, observed in F-VM + 0.5%SS and F-VM + 1%SS. 

### 3.8. Sensory Evaluation

The addition of supplementary materials could significantly increase the organization, taste and flavor of the primary food system, with no difference in color and adhesion. Among all samples, F-VM + 0.5%SS had the most consistent color, while F-VM + 1%SS provided an optimal flavor and the best organization; F-1%SS also tasted the best and had the least residue after swallowing (Figure 7D). Furthermore, F-VM + 0.5%SS obtained a higher sensory total score than F-VM + 1%SS (72.17 ± 4.74 vs. 70.33 ± 3.59) (Table 7). For swallowing ease, F-1%SS and F-VM + 0.5%SS were rated as easy to swallow and the other samples were swallowed normally. However, the swallowing scores of food groups with SS were both significantly higher than those of F and F-VM (*p* < 0.05), which is consistent with the IDDSI test results.

### 3.9. Relationship between Rheology, Texture and Sense 

**Figure 8:**
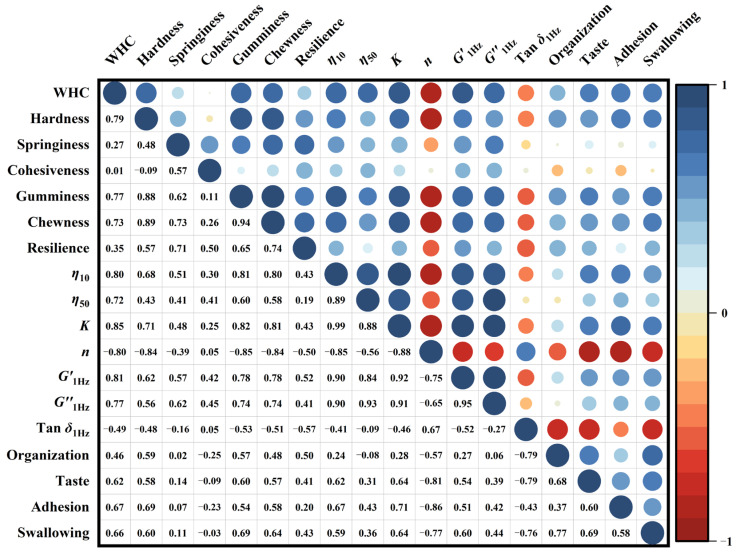
The correlation analysis between rheology, texture and sense of dysphagia food boluses. Blue represents a positive correlation and red represents a negative correlation.

Focusing on the correlation between indexes can be beneficial for the development of dysphagia foods. As shown in Figure 8, the WHC of samples had a mostly strong positive correlation with both textural properties and rheological values (r ⩾ 0.72). This suggests that the WHC could affect the organization and gel properties of samples. The F-1%SS bolus with the best WHC obtained the highest gumminess, chewiness and rheological scores, as well as good taste in the sensory evaluation. This was most likely due to the increased molecular cross-linking, and gel cavities formed to seal in water and strengthen the gel network structure. Similarly, in a study of the effects of psyllium on corn starch properties, the gel rheology and pasting properties were modified because psyllium brought an increase in water-holding capacity [60]. The chewiness, resilience, gel strength and *G*′ of steamed cold noodles were enhanced when the WHC of egg whites on wheat starch gel food was increased from 87% to 94%, relying on the interaction between protein and starch molecules to form a more powerful network [61]. In addition, the texture properties were generally correlated positively with the *K* (0.82 ⩾ r ⩾ 0.25), *G*′ (0.78 ⩾ r ⩾ 0.42) and *G*″ (0.74 ⩾ r ⩾ 0.41), but negatively with *n* (−0.39 ⩾ r ⩾ −0.85). Among the sensory attributes, organization, taste and adhesion were all strongly correlated with hardness (0.69 ⩾ r ⩾ 0.59), chewiness (0.48 ⩾ r ⩾ 0.58) and gumminess (0.60 ⩾ r ⩾ 0.54) in texture indicators, although springiness (0.14 ⩾ r ⩾ 0.02) and cohesiveness (−0.09 ⩾ r ⩾ −0.25) were weakly correlated. It is suggested that supplementary materials affect the viscoelasticity and spatial structure of systems by influencing the contact and interaction between different components in the network, thus affecting the swallowing experiences of food gels. In the preparation of chia seed mucilage-modified soups, the maximum area value and the maximum force value in the texture test were shown to have a strong positive correlation with the oral consistency in swallowing properties [62]. At a lower shear rate of 10 s^−1^, there was a strong relationship between taste (r = 0.62), adhesion (r = 0.67) and apparent viscosity (*η*), indicating that high-viscosity food boluses are less likely to remain in the oral cavity after chewing, with less swallowing-induced malabsorption and discomfort. The Tanδ value, the information about the balance of the viscoelastic modulus, was negatively linked to sensory properties (−0.43 ⩾ r ⩾ −0.79) and swallowing ease evaluation (r = −0.76), suggesting that samples with higher *G*′ and lower *G*″ might be more favored by dysphagia populations. It has been reported that foods with great resistance to deformation (high G* values) and great elasticity (low Tanδ values) could be safe to swallow [6]. Thus, rheological and textural data can be used to provide some indication of the swallowing characteristics of foods. In this study, water-holding capacity, apparent viscosity, springiness, chewiness, consistency and loss modulus were important indicators for regulating food swallowing abilities.

**Figure 9:**
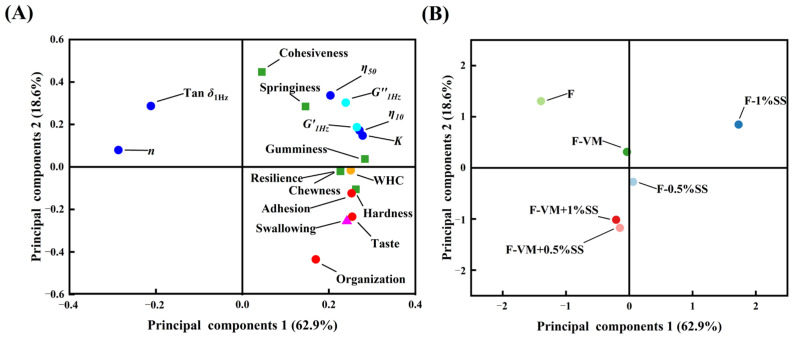
Principal component analysis (loading plot (**A**) and scores plot (**B**)) of dysphagia food with supplementary materials.

In the principal component analysis (PCA) results (Figure 9), PC1 and PC2 explained 81.5% of the total variance, with PC1 accounting for 62.9% and PC2 for 18.6%, respectively. The inter-index relationship analysis in the loading plot (Figure 9A) yielded results consistent with Figure 8, where *G*′_1Hz_, *G*″_1Hz_, *η*_10_, *K*, gumminess, resilience, water holding capacity, hardness and chewiness were strongly positively related with taste, organization and swallowing behavior, while strongly negatively related with *η* and Tanδ. The lower flow behavior index *n* indicates that the sample’s apparent viscosity reduces more quickly with increasing shear rates, which in turn means that the sample has a correspondingly high viscosity at lower shear rates. Since dysphagia patients typically have a lower oral shear rate, chewing higher-viscosity foods helps them eat and replenish nutrients [8]. In the score plot (Figure 9B), the dot of F was visually distant from the other samples, suggesting that the addition of supplementary materials had greater effects on food system properties. VM and SS made a large difference in food groups on PC1. However, VM slowed down the promotion of salt ions on food gels, which was in line with the rheological property results. F-VM + 0.5%SS and F-VM + 1%SS do not differ either on PC1 or on PC2. However, by comparing with F-0.5%SS and F-1%SS, VM exerted a greater effect when SS content was higher, especially on PC2. Therefore, the comprehensive picture showed that the F-VM + 0.5%SS food bolus was more suitable for dysphagia patients, due to its good texture, high total sensory score and appropriate swallowing ease.

## 4. Conclusions

This work demonstrated that four supplementary materials (VM and SS) affected the texture, flow properties and swallowing characteristics of a food bolus, and all samples belonged to the pureed level 4 in the IDDSI framework. SS changed the spatial structure and viscoelasticity of the gel system by enhancing the intermolecular interaction forces, resulting in a more networked gel pattern, which increased the water-holding capacity, viscosity, storage modulus, chewiness and swallowing ability of dysphagia foods. VM could endow the food bolus with richer color characteristics but increased the residues on the spoon, with the attendant risk of accidental aspiration. F-VM + 0.5%SS obtained a better evaluation than F-VM + 1%SS in terms of sensory properties and swallowing ease. In addition, the texture and fluidity of a bolus also showed a strong correlation with swallowing characteristics. The information provided in this work should help to prepare nutritional dysphagia foods, but the mechanism of supplementary materials acting together to influence food swallowing still needs to be explored more deeply.

## Figures and Tables

**Table 1:** The design of the dysphagia food bolus.

Dysphagia Food	PSO	RIS	WPI
A	15%	60%	25%
B		65%	20%
C		70%	15%
D	10%	60%	30%
E		65%	25%
F		70%	20%

**Table 2:** The nutrient requirements of dysphagia food (the additional contents are on the top of the RIS, PSO and WPI total weight).

Supplementary Materials		GB 29922-2013 for People over 10 Years of Age Special Medical Food Requirements for Nutrients/100 kJ	Nutrient Contents in Mixed Vitamins and Mixed Minerals/g	Additional Contents in Food Bolus
Mixed minerals	Mg (mg)	≥4.4	130	1%
	Fe (mg)	0.20–0.55	9.5	
	Zn (mg)	0.1–0.5	6	
	Ca (mg)	≥13	260	
	P (mg)	≥9.6	189	
Mixed vitamins	VA (μg)	9.3–53.8	3376	0.20%
	VB_1_ (mg)	≥0.02	3.906	
	VB_2_ (mg)	≥0.02	3.9	
	VB_6_ (mg)	≥0.02	3.91	
	VB_12_ (μg)	≥0.03	9.8	
	VC (mg)	≥1.3	525	
	VD (μg)	0.19–0.75	4.075	
	VE (mg)	≥0.19	36	
	Nicotinic acid (mg)	≥0.05	62.5	
	Folic acid (μg)	≥5.3	1300	
	Pantothenic acid (mg)	≥0.07	12.512	
Salt		≤5.0 g/d		0.5%, 1%
Sugar		≤10.0 g/d		0.5%, 1%

**Table 3:** The design of dysphagia food with supplementary materials (the additional contents are on the top of RIS, PSO and WPI total weight).

Dysphagia Food	RIS:WPI:PSO	Mixed Minerals	Mixed Vitamins	Salt	Sugar
F	70%:20%:10%	-	-	-	-
F-VM		1%	0.2%	-	-
F-0.5%SS		-	-	0.5%	0.5%
F-1%SS		-	-	1%	1%
F-VM + 0.5%SS		1%	0.2%	0.5%	0.5%
F-VM + 1%SS		1%	0.2%	1%	1%

**Table 4:** Score criteria for the sensory evaluation of dysphagia samples.

Criteria	Standard for Evaluation	Score
Color	Not uniform	0–6
	Generally uniform	7–11
	Very uniform	12–16
Organization	Loose structure, poor elasticity and chewiness.	0–6
	Tight structure, normal elasticity and chewiness	7–11
	Tight structure, good elasticity and chewiness	12–16
Taste	Poor taste and hard texture	0–6
	Average taste and soft texture	7–11
	Good taste and soft texture	12–16
Flavor	Strange smell and unacceptable	0–6
	Light smell and acceptable	7–11
	Good smell and very acceptable	12–16
Adhesion	More oral residue after swallowing	0–6
	Less oral residue after swallowing	7–11
	No residue in oral after swallowing	12–16
Swallowing	Hard to swallow	0–7
	Normal to swallow	8–13
	Easy to swallow	14–20

**Table 5:** Steady flow and dynamic rheological parameters of dysphagia food with supplementary materials.

Sample	F	F-VM	F-0.5%SS	F-1%SS	F-VM + 0.5%SS	F-VM + 1%SS
Flow Sweep						
*η*_0.1_ (Pa·s)	1124 ± 50 ^c^	1740 ± 45 ^b^	1857 ± 85 ^b^	3863 ± 151 ^a^	1850 ± 53 ^b^	1155 ± 16 ^c^
*η*_1_ (Pa·s)	167.2 ± 10.3 ^d^	263.6 ± 6.2 ^b^	257.2 ± 5.2 ^b^	462.7 ± 14.1 ^a^	258.2 ± 3.3 ^b^	192.0 ± 0.8 ^c^
*η*_10_ (Pa·s)	26.70 ± 2.02 ^c^	35.17 ± 0.77 ^b^	33.87 ± 1.15 ^b^	52.20 ± 1.47 ^a^	33.74 ± 0.50 ^b^	25.75 ± 0.14 ^c^
*η*_50_ (Pa·s)	8.437 ± 0.434 ^b^	7.842 ± 0.238 ^b^	8.220 ± 0.450 ^b^	11.39 ± 0.28 ^a^	7.894 ± 0.155 ^b^	6.443 ± 0.025 ^c^
*K* (Pa·s^n^)	168.9 ± 10.2 ^c^	247.7 ± 3.5 ^b^	258.8 ± 9.9 ^b^	459.2 ± 13.7 ^a^	249.3 ± 4.8 ^b^	179.1 ± 2.5 ^c^
*n* (-)	0.225 ± 0.008 ^a^	0.130 ± 0.009 ^c^	0.122 ± 0.005 ^c^	0.055 ± 0.002 ^d^	0.125 ± 0.004 ^c^	0.159 ± 0.005 ^b^
*R*^2^ (Power law)	0.988	0.981	0.992	0.970	0.962	0.982
Frequency Sweep						
*G*′_1Hz_ (Pa)	487.3 ± 26.7 ^d^	598.1 ± 12.6 ^b c^	582.9 ± 4.5 ^b c^	1521 ± 44 ^a^	533.1 ± 10.0 ^c d^	643.7 ± 52.4 ^b^
*G*″_1Hz_ (Pa)	74.13 ± 4.13 ^b^	73.31 ± 2.08 ^b^	79.72 ± 6.72 ^b^	148.0 ± 2.3 ^a^	58.20 ± 3.33 ^c^	62.40 ± 3.06 ^c^
Tan *δ*_1Hz_ (-)	0.159 ± 0.010 ^a^	0.123 ± 0.002 ^b c^	0.137 ± 0.010 ^b^	0.097 ± 0.003 ^d^	0.109 ± 0.004 ^c d^	0.098 ± 0.009 ^d^
*G*′_0_	498.7 ± 22.2 ^d^	613.2 ± 11.6 ^b^	587.3 ± 10.5 ^b c^	1517 ± 41 ^a^	541.4 ± 9.3 ^c d^	648.3 ± 50.8 ^b^
*n*′	0.094 ± 0.005 ^a^	0.075 ± 0.001 ^c^	0.082 ± 0.004 ^b^	0.055 ± 0.002 ^e^	0.072 ± 0.003 ^c^	0.065 ± 0.002 ^d^
*R*^2^ (Power law)	0.990	0.976	0.992	0.985	0.991	0.995
*G*″_0_	74.80 ± 3.83 ^b^	75.31 ± 2.16 ^b^	81.00 ± 5.94 ^b^	152.0 ± 3.5 ^a^	58.94 ± 2.88 ^c^	63.83 ± 3.31 ^c^
*n*″	0.251 ± 0.002 ^b^	0.212 ± 0.002 ^c^	0.148 ± 0.010 ^d^	0.134 ± 0.002 ^e^	0.264 ± 0.007 ^a^	0.267 ± 0.001 ^a^
*R*^2^ (Power law)	0.988	0.994	0.982	0.980	0.996	0.976

Mean values ± standard deviation. For each rheological property, mean values without the same letter in the same row are significantly different (*p* < 0.05). *η*_0.1_, *η*_1_, *η*_10_, *η*_50_ and *η*_100_, apparent viscosities at shear rates 0.1, 1, 10, 50 and 100 s^−1^; K and n, consistency and flow behavior index from the power-law model; R^2^, determination coefficient of power-law model; *G*′_1Hz_, storage modulus at 1 Hz; *G*″_1Hz_, loss modulus at 1 Hz; Tanδ_1Hz_, loss factor at 1 Hz; *G*′_0_ and *G*″_0_, correspond to *G*′ and *G*″ values with frequency (f) at 1 Hz; *n*′ and *n*″, regression coefficients relating *G*′ and *G*″ with frequency (f) in Hz.

**Table 6:** Effect of supplementary materials on the textural features of food boluses.

Sample	Hardness (g)	Springiness	Cohesiveness	Gumminess	Chewiness	Resilience
F	200.9 ± 18.0 ^c^	0.884 ± 0.010 ^ab^	0.672 ± 0.053 ^ab^	134.8 ± 13.1 ^d^	119.0 ± 10.5 ^d^	0.136 ± 0.008 ^c^
F-VM	395.7 ± 19.2 ^b^	0.940 ± 0.010 ^a^	0.647 ± 0.059 ^abc^	275.0 ± 18.3 ^b^	241.3 ± 31.6 ^bc^	0.261 ± 0.069 ^ab^
F-0.5%SS	534.4 ± 10.8 ^a^	0.911 ± 0.027 ^a^	0.556 ± 0.038 ^c^	308.8 ± 21.0 ^b^	271.5 ± 31.4 ^b^	0.204 ± 0.040 ^abc^
F-1%SS	563.5 ± 71.5 ^a^	0.929 ± 0.032 ^a^	0.683 ± 0.040 ^a^	382.0 ± 29.0 ^a^	354.7 ± 28.5 ^a^	0.281 ± 0.033 ^a^
F-VM + 0.5%SS	377.0 ± 21.3 ^b^	0.850 ± 0.037 ^b^	0.571 ± 0.028 ^bc^	217.7 ± 2.4 ^c^	182.5 ± 7.0 ^c^	0.165 ± 0.035 ^bc^
F-VM + 1%SS	392.4 ± 76.1 ^b^	0.884 ± 0.026 ^ab^	0.608 ± 0.033 ^abc^	206.1 ± 5.3 ^c^	211.3 ± 44.1 ^bc^	0.254 ± 0.070 ^ab^

Mean values ± standard deviation. Mean values without the same letter in the same column are significantly different (*p* < 0.05).

**Table 7:** Effect of supplementary materials on the color and sensory parameters of the dysphagia food boluses.

Sample	Chroma			Sensory Test	
	C_ab_*	h_ab_*	ΔE*	Swallowing	Total Score
F	4.300 ± 0.654 ^c^	0.422 ± 0.055 ^a^	-	11.17 ± 2.27 ^c^	54.50 ± 3.04 ^c^
F-0VM	8.335 ± 0.068 ^a^	−1.169 ± 0.010 ^e^	29.84 ± 0.32 ^b^	11.33 ± 1.11 ^c^	66.33 ± 4.64 ^b^
F-0.5%SS	2.465 ± 0.052 ^d^	−0.371 ± 0.080 ^c^	5.325 ± 0.324 ^e^	13.00 ± 1.41 ^ab^	68.67 ± 3.77 ^ab^
F-1%SS	2.079 ± 0.033 ^e^	−0.057 ± 0.031 ^b^	8.364 ± 0.140 ^d^	15.00 ± 1.63 ^a^	73.17 ± 2.48 ^a^
F-VM + 0.5%SS	7.999 ± 0.115 ^a^	−1.048 ± 0.015 ^d^	31.18 ± 1.58 ^a^	14.17 ± 1.67 ^a^	72.17 ± 4.74 ^a^
F-VM + 1%SS	7.174 ± 0.065 ^b^	−1.043 ± 0.020 ^d^	23.11 ± 1.05 ^c^	13.00 ± 1.15 ^ab^	70.33 ± 3.59 ^ab^

Mean values ± standard deviation. Mean values without the same letter in the same column are significantly different (*p* < 0.05). C_ab_*, chroma; h_ab_*, hue; ΔE*, color variations.

## Data Availability

The data used to support the findings of this study can be made available by the corresponding author upon request.
